# Effects of aurantiamide on a rat model of renovascular arterial hypertension

**DOI:** 10.1007/s00424-023-02850-8

**Published:** 2023-08-15

**Authors:** Mutay Aslan, Filiz Basralı, Pınar Ülker, Zerrin Barut, Çağatay Yılmaz, Tuğçe Çeker, Nur Özen, Aleyna Öztüzün, Özlem Elpek

**Affiliations:** 1 Department of Medical Biochemistry, Akdeniz University Faculty of Medicine, Antalya, 07070 Turkey; 2 Department of Physiology, Akdeniz University Faculty of Medicine, Antalya, Turkey; 3 Faculty of Dentistry, Antalya Bilim University, Antalya, Turkey; 4 Department of Pathology, Faculty of Medicine, Akdeniz University, Antalya, Turkey

**Keywords:** Aurantiamide, Hypertension, Endothelial nitric oxide synthase

## Abstract

Asperglaucide (ASP) is an aurantiamide, an effective constituent of purslane (*Portulaca oleracea* L.), a safe to eat greenery. Effects of ASP on endothelial function, endothelial nitric oxide synthase (eNOS) expression, vascular fluidity, renal and vascular reactive oxygen, and nitrogen species (ROS/RNS) production was examined in the two-kidney one-clip (2 K-1C) rat model of renovascular arterial hypertension. ASP toxicity, dose dependent eNOS gene expression and protein levels were also analyzed in human umbilical vein endothelial cells (HUVEC). The 2 K-1C model of hypertension was created via surgery and mean blood pressure (MBP) was measured by tail-cuff method during four weeks of ASP treatment. Erythrocyte deformability was monitored by rotational ektacytometry, while vascular constrictor and dilator responses were determined in organ baths. eNOS gene expression and protein levels were assessed in thoracic aorta and HUVEC. MBP was significantly decreased in hypertensive rats treated with ASP. Endothelium dependent vascular dilator and constrictor responses were also considerably improved following ASP treatment. There was a notable increase in red blood cell deformability in hypertensive rats treated with ASP as compared to hypertensive rats alone. A significant increase was observed in eNOS gene expression and protein levels in both normotensive and hypertensive rats treated with ASP. Treatment of HUVEC with 3 µM ASP notably increased eNOS mRNA and protein levels. In conclusion, ASP lowered blood pressure, improved endothelium-mediated relaxation, decreased renovascular ROS/RNS production in hypertensive rats. ASP also increased eNOS protein expression in aorta and HUVEC at nontoxic doses. ASP may have future potential as an anti-hypertensive agent.

**Supplementary Information:**

The online version contains supplementary material available at 10.1007/s00424-023-02850-8.

## Introduction

Endothelium covers the internal layer of all blood vessels. It creates an active biological fence between blood and tissues. This barrier plays an important adaptive role in vascular tension, in regulating vascular permeability, inflammatory reactions, and in harmonizing fibrinolysis/coagulation [31]. Any alteration in the morphology and function of the endothelium leads to failure of regular physiological tasks. This situation is described as endothelial dysfunction [44]. In the dysfunctional endothelium, the barrier function is damaged, vasomotion is impaired due to an increase or decrease in vasoactive factors, and prothrombotic/procoagulant activity increases [44]. The most used method in the investigation of endothelial dysfunction is the evaluation of endothelial nitric oxide-dependent functions. The resulting change in endothelium-dependent vascular tone is an indication of further alterations of the endothelium. The equilibrium between endothelial-derived contraction and relaxant factors supports vascular homeostasis. Once this equilibrium fails, vascular inflammation, leukocyte adhesion, platelet activation, predisposition to thrombosis, impaired coagulation, pro-oxidative changes and vasoconstriction occurs [44]. Endothelial dysfunction is the initial sign of vascular disorders and is observed in numerous pathological situations. Endothelial malfunction relates to disorders having higher cardiovascular risk such as hyperlipidemia, atherosclerosis, systemic inflammation, obesity, diabetes, hypertension, and homocysteinemia [44]. Endothelial dysfunction, especially decreased vascular endothelial-derived nitric oxide, has been investigated in many experimental and clinical studies as a possible mechanism for the detrimental vascular effects of hypertension. Previous studies have reported a decrease in eNOS protein in the 2 K-1C rat hypertension model [25]. Renal blood flow decreases due to renal artery stenosis in the 2 K-1C model and thus, the renin–angiotensin–aldosterone system (RAAS) is activated. With the activation of RAAS, water and electrolyte reabsorption in the kidney increases, thereby increasing blood pressure [25]. Renovascular hypertension develops in this model of hypertension.

Knowledge of the process causing endothelial dysfunction in hypertension has greatly progressed lately. Macrovascular complications of hypertension such as coronary dysfunction, myocardial infarction, stroke and augmented arterial rigidity relate to endothelial dysfunction, which are most likely or at least in part attributable to insufficiency of the antiatherogenic and vasoprotective effects of endothelial-derived nitric oxide [16]. Recent studies on endothelial dysfunction support its clinical importance in hypertension and have led to important concepts regarding the pathophysiology of the disease [48]. These findings suggest that aiming at endothelial dysfunction, especially decreased nitric oxide presence, may generate beneficial effects in hypertensive patients. This concept needs further investigation in experimental studies. The effect of ASP on endothelial function in experimental hypertension has not been investigated and its efficacy in this model is unknown, highlighting the uniqueness of the study in developing potential therapeutic targets.

ASP is an aurantiamide that can be isolated from P. aurantiacum and has anti-inflammatory, antibacterial, antioxidant, and anticancer properties [42]. Aurantiamide and aurantiamide acetate are the major effective constituents of purslane (*Portulaca oleracea* L.), a safe to eat greenery with a variety of biological actions [14]. Antihypertensive effects of purslane extracts have been reported in an animal model of hypertension [43]. Aurantiamide has a dipeptide structure and consists of N-benzoylphenylalanine and phenylalanine [9]. Moringa Genus, Black Soybean and Adzuki Bean containing Aurantiamide Acetate have also been reported to have blood pressure lowering properties [24].

ASP inhibits the production of inducible nitric oxide synthase (iNOS) in microglia cells stimulated with lipopolysaccharide (LPS) [53]. In contrast, the effects of ASP on eNOS, vascular fluidity and vascular endothelial function are unknown. We hypothesized that a regulatory effect on vascular tone and endothelial function could be achieved by administering ASP in a hypertensive rat model.

## Materials and methods

### Animals and induction of 2K1C hypertension

All experimental protocols conducted on rats were executed in agreement with the principles set up by the Institutional Animal Care and Use Committee at Akdeniz University (Decision No:153-Date: 11.11.2019). Forty normotensive male albino Wistar rats aged 1 month, weighing 180–240 g was housed in stainless steel cages in groups of four rats per cage and given food and water ad libitum. Animals were kept in 12 h light–dark cycles and a regular temperature. Animals were accommodated in well-ventilated rooms with a relative humidity of 40–60% and an ambient temperature of 20–24 °C. Rats were randomly divided into 5 groups of 8 animals including: control (C); sham operated (sham); asperglaucide-treated (ASP); hypertensive (HT); and asperglaucide-treated hypertensive (HT + ASP).

Hypertension was induced with a 2 K-1C Goldblatt model [26]. The right kidney was not operated, and the left renal artery was narrowed to 0.22 mm in diameter. The rats were anesthetized intraperitoneally with 90 mg/kg ketamine and 10 mg/kg xylazine before the operation. The abdomen was shaved and disinfected with betadine. After disinfection, a midline incision was made, and the left kidney was exposed. The left renal artery was isolated from the renal vein and the circumference of the left renal artery was gently narrowed to 0.22 mm in diameter with 5/0 non-absorbable silicone-coated silk suture (Boz Medical Materials Industry and Trade Inc., Ankara, Turkey) to induce 2 K-1C renovascular hypertension. The same procedure was applied to sham group rats, but the left renal artery was not narrowed. After surgery, the peritoneum was sutured with 3/0 absorbable polyglycan suture and the skin layer was sutured with 3/0 non-absorbable silk suture. Hypertensive state was confirmed by blood pressure measurements and was maintained for 4 weeks**.** The mean blood pressure of rats was measured by using a noninvasive tail-cuff method. Arterial blood pressure measurements were taken weekly at the same time of day (between 9:00 a.m. and 12:00 noon) to prevent the effect of the circadian cycle. After each animal was received, their basal blood pressure was measured, and regularly recorded every week for 4 weeks until sacrificed. The data acquisition system reported blood pressure (mmHg) as systolic, diastolic, and average. Each measurement was repeated three times and the three data were averaged. Data were obtained with a MAY-BPHR 9610-PC unit and MP 150 data-acquisition system (BIOPAC Systems; Santa Barbara, CA).

### Asperglaucide treatment

A 100 mM stock was prepared by dissolving 10 mg of ASP (Toronto Research Chemicals, ON, Canada. Catalogue number: A780120) in 225 µl of DMSO. ASP was administered intraperitoneally (i.p.) for 4 weeks, 0.5 mg/kg daily i.p. injections for 5 days each week, with a total weekly dose of 2.5 mg/kg. A total of 10 mg/kg of ASP was given to each animal. Control, sham-operated and hypertension group rats were injected with an equivalent volume (100 µl) of i.p. DMSO. The selected ASP dose and method of preparation were determined according to previous studies [42]. After four weeks of treatment, animals were administered 45 mg/kg of ketamine and 10 mg/kg of xylazine intraperitoneally. Rats were sacrificed by exsanguination and tissues were collected.

### Measurement of serum biochemistry

Two mL blood samples were collected into tubes without an anticoagulant, for biochemical analyses. Blood samples were centrifuged at 3000 rpm for 10 min at room temperature to obtain serum. Aspartate aminotransferase (AST), alanine aminotransferase (ALT), albumin (ALB), total bilirubin (TBIL), creatinine (CRE) and blood urea nitrogen (BUN) were assayed using Fujifilm kits and analyzed on Fujifilm DRI-CHEM NX500 (FUJIFILM Co.,Tokyo, Japan).

### Measurement of vasodilatation and vasoconstriction responses

The thoracic aorta was removed and kept on ice in Krebs solution (110 mM NaCl, 5 mM KCl, 24 mM NaHCO_3_, 1 mM KH_2_PO_4_, 1 mM MgSO_4_7H_2_O, 2.5 mM CaCl_2_, 10 mM glucose, 0.02 mM EDTA; pH = 7.4). After the tissue was washed with cold Krebs, fat and connective tissue were removed from the artery at the appropriate magnification using a dissecting microscope (SZ61, Olympus, Japan). The isolated thoracic aorta was divided into rings of appropriate length. The rest of the aorta tissue was prepared for histological and biochemical studies.

Vascular tone was analyzed as previously [34]. Isolated thoracic aorta was cut into 2–2.5 mm long pieces and aortic rings were suspended between two stainless hooks, placed in an organ bath containing 10 ml of Krebs solution at 37 °C (pH 7.4) gassed with a mixture of 95% O_2_—5% CO_2_. The upper hook was connected to an isometric transducer [Force Displacement Transducer (FDT) 10-A, MAY, Ankara, Turkey]. Rings were stretched at 1 g resting tension for a total of 60 min and the bath solution was changed every 15 min. The 1 h rest period was followed by vitalization via adding Krebs solution containing 20 nM KCl and 10^–7^ M phenylephrine (Sigma, St. Louis, USA). This process was repeated 3 times in succession. After the vitalization procedure, the vessels were washed with Krebs solution and an additional 30-min rest period was started. The presence of a functional endothelium was confirmed by the ability of acetylcholine (Ach; 10^–6^ M) (Sigma, St. Louis, USA) to produce relaxation of vessels pre-contracted with phenylephrine (Phe; 10^–6^ M). Vessels with a relaxation response of 60% or more were considered as undamaged, and those below 60% were excluded from the study. The contraction and relaxation signal responses from the vessels were amplified by an amplifier (Transducer Amplifier #GTA0303, MAY, Ankara, Turkey) and processed through the MP150 data acquisition device (BIOPAC SYSTEMS Inc., Goleta, CA, USA) and software program (AcqKnowledge Data Acquisition and Analysis Software version 3.8.2). Contraction responses were examined by adding increasing doses of KCl (20–80 mM) or Phe (10^–9^–3 × 10^–5^ M) to the bath solution. Endothelium-dependent or -independent relaxation response protocols were carried out by adding ACh (10^–9^–3 × 10^–5^ M) or sodium nitroprusside (SNP; 10^–10^– 3 × 10^–4^ M), respectively.

### Red blood cell deformability measurements

Red blood cell (RBC) deformability was measured at 0.3–50 Pascal (Pa) shear stress by laser diffraction analysis via an ektacytometer (LORCA, RR Mechatronics, Hoorn, The Netherlands) as described previously [46].

### Tissue immunohistochemical staining

Aortic tissues were fixed in 10% buffered formalin solution, rinsed in phosphate-buffered saline (pH 7.4), embedded in paraffin, and cut into 4-µm sections. Sections were deparaffinized in xylene, rehydrated in a decreasing gradient of ethanol and finally rinsed in distilled water. Immunohistochemical staining was performed with the Dako Omnis closed system immunohistochemical staining device (Agilent Technologies, Santa Clara, USA). Working kits used in this system are EnVision FLEX, high pH (Agilent Technologies, Glostrup, Denmark); EnVision FLEX peroxidase-blocking reagent (Dako Omnis); EnVision FLEX/HRP (Dako Omnis); EnVision FLEX DAB + chromogen (Dako Omnis); EnVision FLEX substrate buffer (Dako Omnis) and EnVision FLEX target retrieval solution, high pH (50x) (Dako Omnis). Primary antibody administration was carried out with rabbit polyclonal anti-eNOS (anti-NOS3) (1:50 dilution, #MBS8242404, MyBiosource Inc., San Diego, CA, USA) at 25 °C for 60 min. Biotin-labeled secondary antibody was used and the reaction was carried out with strepdoavidin-peroxidase conjugate. Tissue staining was quantified by morphometric analysis as previously studied [55].

### Western blot analysis

Aortic tissue was homogenized and 6 × 10^6^ HUVEC cells were sonicated in PBS buffer containing protease inhibitor cocktail (Sigma-Aldrich, St. Louis, MO, USA). Supernatants were stored at -80 °C until analyzed. Western blot analysis was performed essentially as described in detail previously [8]. Rabbit polyclonal anti-eNOS (1:200 dilution, #MBS8242404, MyBiosource Inc., San Diego, CA, USA) and anti-actin (1:1000, #AANO1, Cytoskeleton Inc. Denver, CO, USA) for 1 h at room temperature. Horseradish peroxidase–conjugated goat anti-rabbit IgG (1:10,000 dilution; Zymed Laboratories, San Francisco, CA) was used as a secondary antibody, and immunoreactive proteins were visualized by chemiluminescence via ECL reagent (Amersham Pharmacia Biotech, Buckinghamshire, UK). All Western blots were quantified by densitometric analysis using NIH ImageJ 1.44p software.

### Quantitative PCR analysis

Total RNA extraction from rat aortic tissue and HUVEC cells was performed using AxyPrep Multisource Total RNA Miniprep Kit (Axygen Biosciences, CA, USA) according to manufacturer instructions. Purified RNA concentration was determined as described in detail previously [1].

Online Mendelian Inheritance in Man (OMIM), Mouse Genome Informatics and Ensembl databases were used as sources for mRNA sequences. Primers and probes were designed using Oligoware 1.0 software as previously [52] and synthesized by Metabion International AG (Steinkirchen, Germany). The primers and probes used in the study are as shown in Table 1. Real-time PCR analysis was performed as described in detail previously [1]. For each sample, 10 μl of DNAse 1 (Serva, Germany) was added onto 90 μl of RNA and incubated at 38 °C for 15 min, thus making the RNA ready for use. A total of 20.60 µl of sample mix was prepared for determining 18sRNA in rat aortic tissues [10 µl one-run mix (dNTP, DNA polymerase, reverse transcriptase and 2.5 mM MgCl2) 10 µl RNA sample, 0.40 µl (10 pmol) primer mix and 0,20 µl probe (10 pmol)]. Rat vascular tissue eNOS mRNA was determined in a total of 21.60 μl sample mix [10 μl of one-run mix, 10 μl of RNA sample, 1 μl (10 pmol) of primer mix and 0.60 μl of probe (10 pmol)]. β-Actin and eNOS mRNA in HUVEC cell lysates were determined in a total of 21.90 μl of sample mix, containing 10 μl of one-run mix, 10 μl of RNA sample, 1.10 μl (10 pmol) of primer mix and 0.80 μl of probe (10 pmol).

**Table 1:** Primers and probes used for real-time-PCR analysis

Gene accession		
Human ACTB OMIM-102630	Forward Primer	5′-CCC AGC ACA ATG AAG ATC AAG ATC-3′
	Reverse Primer	5′-GGG TGT AAC GCA ACT AAG TCA TAG TC-3′
	Probe	5′-AGA TCA TTG CTC CTG AGC GCA AG-3′
Human Enos OMIM-163729	Forward Primer	5′-GTG GCT GTC TGC ATG GAC CTG-3′
	Reverse Primer	5′-TCC ACG ATG GTG ACT TTG GCT AG-3′
	Probe	5′-CGG ACC TCG TCC CTG TGG-3′
Rat 18 s RNA Ensembl-CT010467.1	Forward Primer	5′-CGG ACA GGA TTG ACA GAT TGA TAG-3′
	Reverse Primer	5′-GTC TCG TTC GTT ATC GGA ATT AAC-3′
	Probe	5′-CTC GAT TCC GTG GGT GC-3′
Rat eNOS MGI-97362	Forward Primer	5′-CTG CTG CCC GAG ATA TCT TCA G-3′
	Reverse Primer	5′-GTG AGT CAG CCC TGG TAG TAA TTG-3′
	Probe	5′-CAG CTG GAA GCG CCA GAG GTA CC-3′

*OMIM* Online Mendelian Inheritance in Man; *MGI* Mouse genome ınformatics; *ACTB* Actin beta; *eNOS* Endothelial nitric oxide synthase

The RT-PCR assay was performed using Mx3000p Multiplex Quantitative PCR system (Stratagene, CA, USA). Cycling conditions were: step 1, 15 min at 42 °C; step 2, 25 s at 96 °C; step 3, 5 s at 96 °C, step 4, 40 s at 60 °C. A total of 45 and 50 cycles were required for rat aorta and HUVEC samples, respectively. The log-linear phase of amplification was monitored to obtain Ct values for each RNA sample using MxPro QPCR Software developed for the Mx3000p analysis system. All reactions were repeated three times and rat vessel eNOS results were normalized to 18sRNA; HUVEC eNOS results were normalized to beta actin. The mRNA levels of each gene were expressed as fold change 2^−(ΔΔct)^. The relative change in the mRNA levels of the genes studied were calculated with respect to control group.

### Measurement of nitrite/nitrate levels

Nitrite/nitrate levels were measured in rat vascular tissue homogenates and HUVEC cell culture media using a fluorometric assay kit (Cayman Chemical, Cat. no. 780051, Ann Arbor, MI, USA) as previously described [7].

### Measurement of reactive oxygen and nitrogen species

Reactive oxygen and nitrogen species were measured by OxiSelect ROS/RNS assay kit (Cell Biolabs, Inc. San Diego,CA, USA) as previously described [3]. The method uses the fluorogenic probe dichlorodihydrofluorescin DiOxyQ (DCFH-DiOxyQ) specific for ROS/RNS. ROS and RNS species react with DCFH, which is rapidly oxidized to the highly fluorescent 2’, 7’-dichlorodihydrofluorescein (DCF). Right kidney and vessel tissue samples were homogenized in cold PBS (10 mg/mL) and centrifuged at 10,000 × g for 5 min. Samples were measured fluorometrically against a DCF standard by using a fluorescence microplate reader (Synergy Mx, Bio-Tek Instruments Inc. Vermont, USA). Free radical content in samples was determined by a DCF standard curve and expressed as DCF nM per mg/ml tissue protein.

### Measurement of total antioxidant capacity

Total antioxidant capacity was measured by OxiSelect Total Antioxidant Capacity Assay Kit (Cell Biolabs, Inc. San Diego,CA, USA) as previously described [3]. Right kidney and vessel tissue samples were homogenized in cold PBS and centrifuged at 10,000 × g for 10 min at 4 °C. The method is founded on the reduction of copper (II) to copper (I) by antioxidants such as uric acid. Upon reduction, the copper (I) ion further reacts with a chromogenic reagent that gives an absorbance at 490 nm. The net absorbance values of antioxidants were compared with a known uric acid standard curve. Results were given as µM uric acid equivalents (UAE) per mg/ml tissue protein.

### Cell culture and treatment conditions

Human umbilical vein endothelial cell line (HUVEC; ATCC® CRL-1730™) was obtained from American Type Culture Collection (ATCC; Manassas, Virginia, USA). Cells were cultured in 1X Kaighn's Modified Ham's F-12 Medium (F-12 K Medium; Gibco, Life Technologies Limited, Paisley, UK). The growth medium was supplemented with the following components: fetal bovine serum (FBS; Gibco, Life Technologies Corporation, Paisley, UK) at a final concentration of 10% (v/v) in 500 ml medium; heparin (Sigma-Aldrich, St. Louis, MO, USA) at a final concentration of 0.1 mg/mL; endothelial cell growth supplement (ECGS; Fisher Scientific, Fair Lawn, New Jersey, USA) at a final concentration of 30 µg/ml; 1% penicillin–streptomycin (Gibco, Life Technologies, Grand Island, NY, USA). Cultures were maintained at 37 °C in a humidified atmosphere of 95% air and 5% CO_2_. Cell lines were grown in suspension culture and were transferred into new flasks after 80% confluence. Cell flasks from which cells were harvested for western-blot and PCR analysis were coated with poly-L-lysine (2 µg/cm^2^) before cells were seeded. A 100 mM stock of ASP was prepared by dissolving 10 mg in 225 µl of DMSO. In most of the experiment’s cells were treated with 3.125 and 6.25 µM ASP for 18 h.

### Cell viability assay

Cell viability was measured colorimetrically using 3-(4,5-dimethylthiazol-2-yl)-2,5-diphenyl tetrazolium bromide (MTT; Gold Biotechnology Inc., St. Louis, MO, USA). MTT was dissolved in PBS and sterile filtered via a syringe filter with a 0.22 µm pore size. Cells were grown to confluence in 96-well plates and incubated with 1 µl/ml DMSO or 3.125–25 µM ASP. Control cells were prepared in plates containing only medium. At the end of the incubation period, MTT was added to each well and the experimental protocol was performed essentially as described in detail previously [13]. Formazan formation is slow in HUVEC cells, so the 96-well plate was incubated at 37 °C, 5% CO_2_ for 24 h.

### Immunofluorescent staining

8-well chamber slides (Merck Millipore, Cork, Ireland) were coated with poly-L-lysine at 2 µg/cm^2^ (#0403, ScienCell Research Laboratories, Carlsbad, CA). Cells were plated at a density of 100.000 cells per chamber and the chamber slide was kept in an incubator at 37 °C, 95% air, 5% CO_2_ overnight in order to achieve 70% confluency. At the end of the incubation period, the medium was removed, and cells were washed twice with 0.01 M cold PBS. The immunofluorescent staining protocol was performed essentially as described in detail previously [13]. Rabbit polyclonal anti-eNOS (anti-NOS3) (1:100 dilution, #MBS8242404, MyBiosource Inc., San Diego, CA, USA) was used as primary antibody and incubated overnight at 4 °C. The secondary antibody, Alexa Fluor-488 conjugated goat anti-rabbit (1:300, # A11008 life Technologies, USA), was applied for 45 min at room temperature and nuclei are counterstained with DAPI (Vector Laboratories Inc., Burlingame, CA, USA) in all experiments. The fluorescence intensity of slides viewed under a microscope (Olympus IX81 fully automated, Tokyo, Japan) was quantified essentially as described in detail previously [13].

### Protein Measurements

Protein concentrations were measured at 595 nm by a modified Bradford assay using Coomassie Plus reagent with bovine serum albumin as a standard (Pierce Chemical Company, Rockford, IL).

### Statistical analysis

Statistical analysis was performed using SigmaStat statistical software version 3.5 (Sigma, St. Louis, MO, USA) or GraphPad Prism Software program for windows version 5.03. as described in detail previously [13]. Statistical analysis for each measurement is described in figure legends.

## Results

### Blood pressure

The surgical procedure of creating the 2 K-1C renovascular hypertension are shown in supplementary Fig. 1A-1 and  A-2. Representative photographs of rat kidneys and changes in kidney weight four weeks after induction of 2K1C hypertension are shown in supplementary Fig.  1B and C, respectively.

**Fig. 1:**
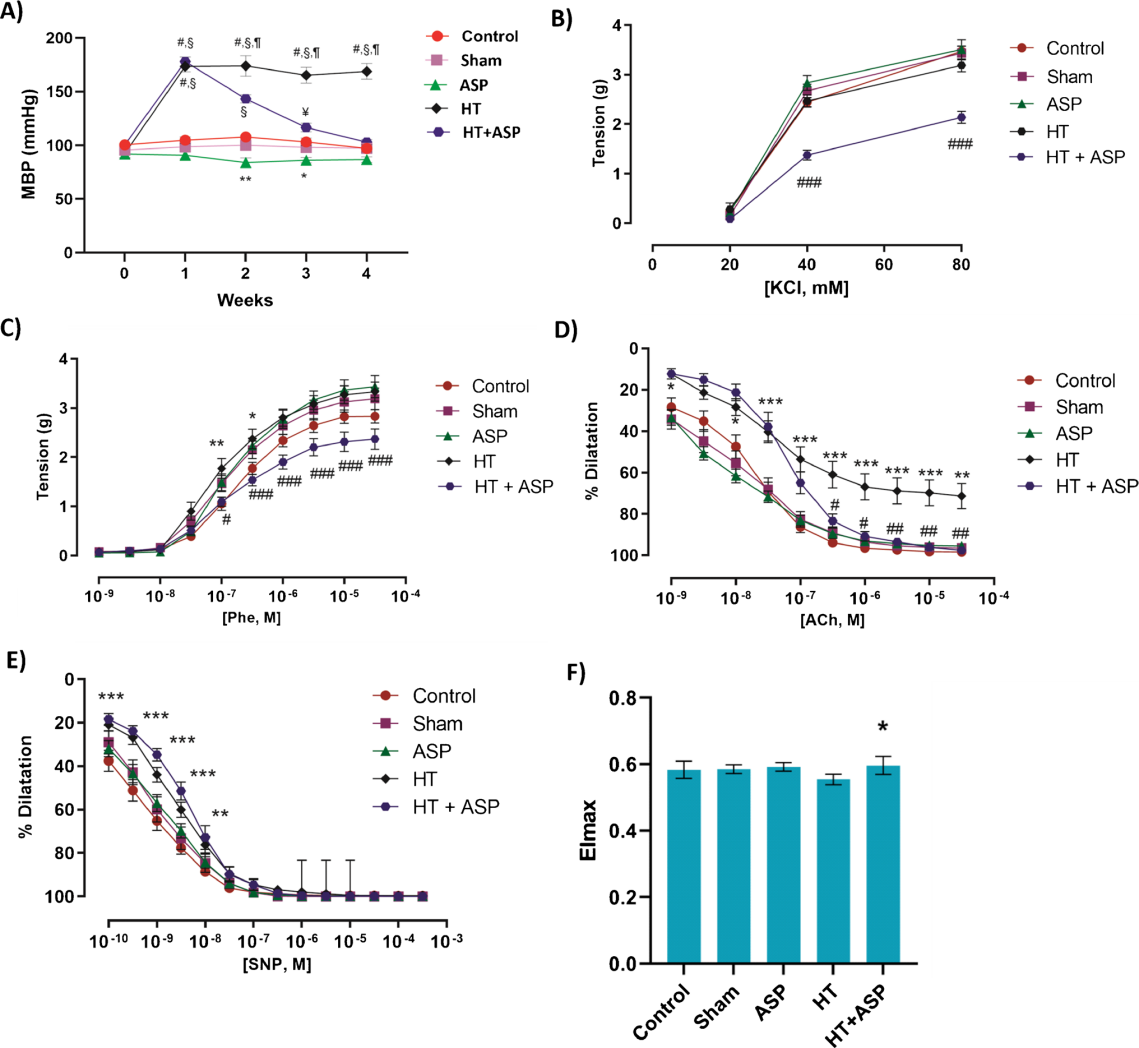
**A)** Mean arterial blood pressure (MBP). ASP, asperglaucide-treated; HT, hypertension. Data are representative of 8 separate experiments and values are given as mean ± SEM. Statistical analysis was done via two-way ANOVA and difference between groups was determined by Tukey multiple comparison test. #*p* < 0.001, compared with week 0 values within the same group. §p < 0.001, compared with control, sham and ASP groups. ¶*p* < 0.01 compared with the HT + ASP group. ¥, *p* < 0,001 compared with the ASP group. **p < 0.05, compared with control and sham groups. *p < 0.05, compared with control group. **B)** Evaluation of the vascular responsiveness and cumulative dose–response curves of isolated aortic rings. Values are given as mean ± SEM (*n* = 8). ASP, asperglaucide-treated; HT, hypertension. Dose–response curves were evaluated statistically using two-way ANOVA followed by Bonferroni post-hoc test. Vasoconstriction responses to KCl. ### *p* < 0.001 HT + ASP vs. all other groups. **C)** Vasoconstriction responses to phenylephrine (Phe). *p < 0.05, **p < 0.01, HT vs. control. #*p* < 0.05, ###*p* < 0.001, HT + ASP vs. HT. **D)** Vasorelaxation responses to acetylcholine (ACh). *p < 0.05, **p < 0.01, ***p < 0.001, HT vs. control. #*p* < 0.05, ##*p* < 0.01, HT + ASP vs. HT. **E)** Vasorelaxation responses to sodium nitroprusside (SNP). **p < 0.01, ***p < 0.001, HT and HT + ASP groups vs. control. **F)** RBC maximal elongation indexes (EImax). Data are representative of **7–8** separate experiments and values are given as mean ± SD. One-way ANOVA and Tukey's posthoc test were used for statistical analysis. *p < 0.05, compared with the HT group

Mean arterial blood pressure values are shown in Fig. 1A. Baseline (week 0) MBP did not significantly differ between groups (*p* > 0.05). No significant difference was observed between control and SHAM groups throughout the study (*p* > 0.05). The 2nd week MBP was significantly lower in the ASP group compared to control and SHAM groups (**, *p* < 0.05). The 3rd week MBP in the ASP group was significantly lower than the control group only (*, *p* < 0.05). When the HT group was evaluated within itself, MBP measured at 1st, 2nd, 3rd and 4th weeks after surgery was significantly higher compared to week 0 (#, *p* < 0.001). When the HT group was compared with other groups, MBP at 1st, 2nd, 3rd and 4th weeks were significantly higher compared to control, SHAM and ASP groups (§, *p* < 0.001). There was no significant difference in blood pressure between HT and HT + ASP groups at 1st week after surgery (*p* > 0.05). The initiation of ASP treatment in hypertensive animals (HT + ASP group), caused a significant decrease in MBP levels in the 2nd, 3rd and 4th weeks when compared to the HT group (¶, *p* < 0.001). The 2nd week MBP in the HT + ASP group was significantly higher compared to control, SHAM and ASP groups (§, *p* < 0.001). The 3rd week MBP in the HT + ASP group was found to be significantly higher than the ASP group only (¥, *p* < 0.001).

### Changes in body weight

The body weight of each animal was recorded before surgery (week 0; pre-op) and after the 4th week. Results are given in supplementary Fig. 1D. Body weight measured after the 4th week showed a significant increase in all experimental groups, compared to week 0. There was no significant difference between the groups. We have also shown the safety of ASP by examining long-term treatment (10 weeks) effects of vehicle (*n* = 3) vs. ASP (*n* = 3). The body weight of each animal was recorded before treatment (week 0) and after the 10th week. Results are given as supplementary Fig. 2. Body weight measured after the 10th week showed a significant increase in both control and ASP treated rats, compared to week 0. There was no significant difference between the two groups.

### Laboratory values in experimental groups

Serum biochemistry results are given in Table 2. No significant difference was found between the groups. Serum biochemical measurements were also carried out to determine the safety of ASP in long-term treatment (10 weeks). Serum biochemistry results measured at 10 weeks are given as supplement Table 1. No significant difference was found between control and ASP treated rats.

**Table 2:** Laboratory values in experimental groups

	Control (*n* = 8)	Sham (*n* = 8)	ASP (*n* = 8)	HT (*n* = 8)	HT + ASP (*n* = 8)
AST (U/l)	128.50 ± 80.10	169.88 ± 57.26	163.00 ± 43.48	202.00 ± 82.45	135.13 ± 40.65
ALT (U/l)	45.88 ± 6.13	51.50 ± 4.81	47.75 ± 5.44	52.38 ± 4.72	46.63 ± 6.07
ALB (g/dl)	3.53 ± 0.25	3.38 ± 0.18	3.36 ± 0.18	3.44 ± 0.64	3.55 ± 0.16
TBIL (mg/dl)	0.24 ± 0.08	0.23 ± 0.05	0.22 ± 0.05	0.35 ± 0.14	0.30 ± 0.11
CRE (mg/dl)	0.19 ± 0.01	0.19 ± 0.01	0.20 ± 0.01	0.25 ± 0.04	0.24 ± 0.05
BUN (mg/dl)	23.86 ± 1.89	23.14 ± 3.34	21.76 ± 3.77	29.13 ± 4.00	30.04 ± 5.93

Values are given as mean ± SD. *ASP* Asperglaucide-treated; *HT* Hypertension; *ALT* alanine aminotransferase; *AST* Aspartate aminotransferase; *ALB* Albumin; *TBIL* Total bilirubin; *CRE* Creatinine; *BUN* Blood urea nitrogen. Statistical analysis was performed by one-way ANOVA analysis and no significant difference was found between groups

### Vasoconstriction and vasorelaxation responses

KCl dose–response curves (Fig. 1B) at 20-, 40- and 80-mM concentrations showed no significant difference between control, sham, ASP and hypertension groups (*p* > 0.05). However, the contraction responses of the HT + ASP group at increasing KCl concentrations were found to be significantly lower compared to all other groups (###, *p* < 0.001). Phe dose–response curves (Fig. 1C) at increasing concentrations of Phe showed no significant difference between control, sham and ASP groups (*p* > 0.05). Phe-mediated contraction responses were significantly increased in the HT group compared to the control group (*, *p* < 0.05 and **, *p* < 0.01). Contractile responses in the HT + ASP group were significantly reduced compared to the HT group (#, *p* < 0.05 and ###, *p* < 0.001). ASP treatment resulted in improvement in Phe-mediated contractile responses, which were impaired in the HT group.

Acetylcholine dose–response curves (Fig. 1D) at increasing concentrations of ACh showed no significant difference between control, sham and ASP groups (*p* > 0.05). A statistically significant decrease was observed in the vasodilation response to ACh in the HT group compared to the control group (*, *p* < 0.05; **, *p* < 0.01 and ***, *p* < 0.001). There was an improvement in ACh-mediated relaxation responses in the HT + ASP group compared to the HT group (#, *p* < 0.05 and ##, *p* < 0.01). Sodium nitroprusside dose–response curves (Fig. 1E) at increasing concentrations of SNP showed no significant difference between control, sham and ASP groups (*p* > 0.05). A significant decrease was observed in the smooth muscle-mediated relaxation response in the HT and HT + ASP groups compared to the control group (**, *p* < 0.01 and ***, *p* < 0.001). No difference was found in the SNP response in the HT + ASP group compared to the HT group (*p* > 0.05).

### RBC deformability

Erythrocyte deformability results are given in Fig. 1F. The EImax values showed no significant difference between control, sham and ASP groups (*p* > 0.05). The EImax value decreased in the HT group compared to control, however this decrease was not significant (*p* = 0.051). There was a significant increase in erythrocyte deformability in the HT + ASP group compared to the HT group (*, *p* < 0.01). This suggests that there was an increase in the ability of erythrocytes to change their shape as they pass through vessels, and an increase in blood fluidity was achieved.

### Aorta eNOS protein and mRNA levels

**Fig. 2:**
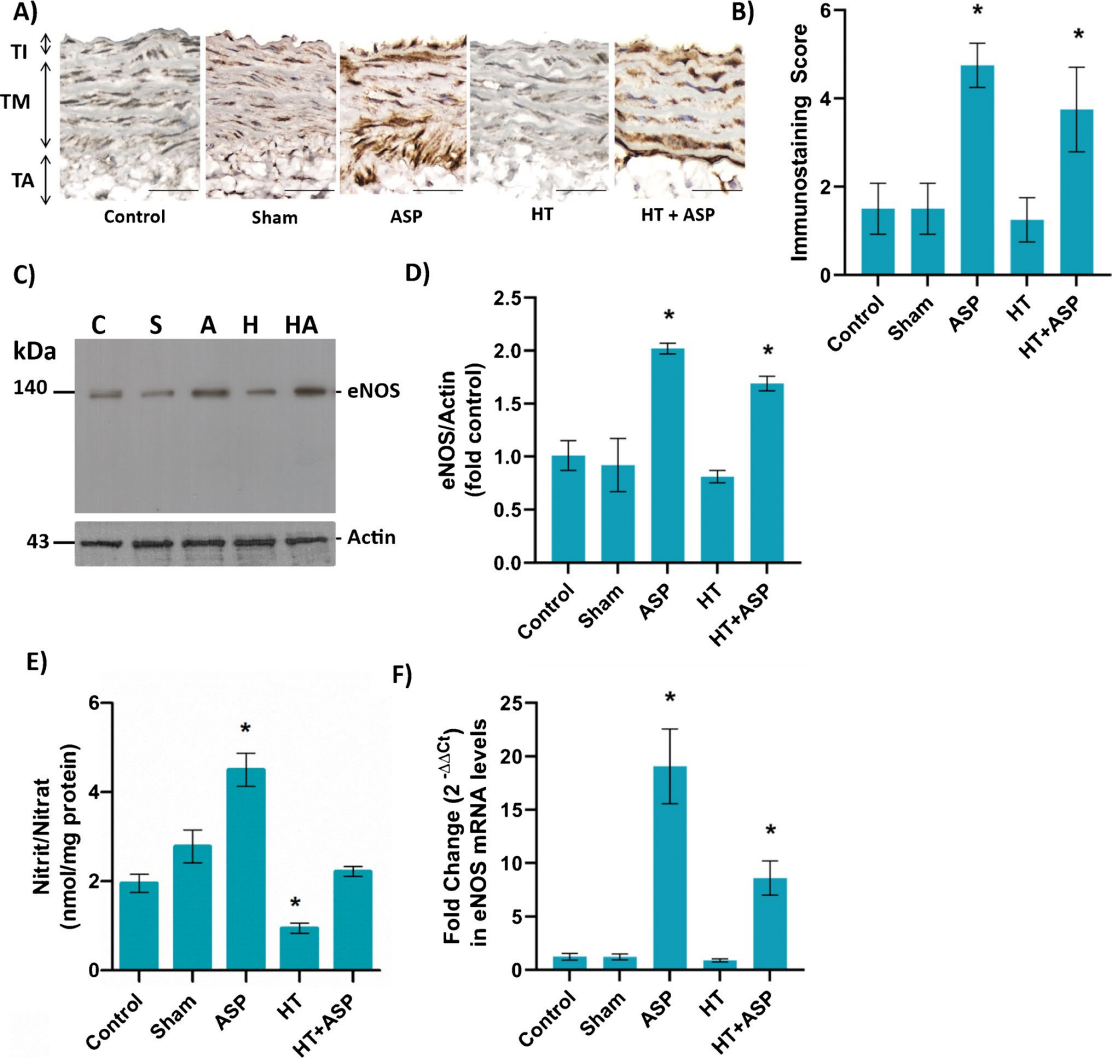
**A)** Representative endothelial nitric oxide synthase (eNOS) immunoperoxidase staining in descending thoracic aortic segments. TI, tunica intima; TM, tunica media; TA, tunica adventitia. Scale bar 100 μm. ASP, asperglaucide-treated; HT, hypertension. Tunica intima includes endothelial cells and inner elastic lamina. Tunica media is the thickest layer. It contains multiple layers of elastic collagen fibers, smooth muscle cells and the outer elastic lamina. Tunica adventisia contains fibro-elastic tissue. Aortic segments from ASP treated rats display intense immunoperoxidase staining for eNOS (dark brown) that is associated with the endothelium and, to a lesser extent, smooth muscle cells. **B)** eNOS immunostaining score. Values are mean ± SD. *n* = 4. Statistical analysis of eNOS staining scores was performed by one-way ANOVA analysis followed by Tukey test. *p < 0.01, vs. control, sham and HT groups. **C)** Western blot analysis of eNOS in aortic tissue. C, control; S, sham; A, ASP-treated; H, hypertension; HA, hypertension + ASP-treated. **D)** The band density of blots was determined by ImageJ software. Data shown are representative of 3 separate measurements and values are given as mean ± SD. Statistical analysis was performed by one way ANOVA and all pairwise multiple comparison procedures done by Tukey test. *p < 0.001, vs. control sham and HT groups. **E)** Nitrite/Nitrate levels of aortic tissue. Data shown are representative of 7–8 separate experiments and values are shown as mean ± SEM. Statistical analysis was performed by one-way ANOVA with all pairwise multiple comparison procedures done by Dunnet’s T3 multiple comparison test. *p < 0.05, vs. control, sham and HT + ASP groups **F)** Aortic eNOS mRNA levels. Data are representative of 8 separate experiments and values are given as mean ± SEM. Statistical analysis was done with Kruskal Wallis one-way analysis of variance and Tukey's posthoc test was used for comparison between groups. *p < 0.05, vs. control, sham and HT groups

Endothelial NOS protein staining in aortic tissue is shown in Fig. 2A. Endothelial NOS protein is stained red brown in endothelial cells located in the tunica intima and in the tunica media. The immunostaining scores determined in the experimental groups are shown in Fig. 2B. ASP administration significantly increased eNOS protein levels compared to control, sham and HT groups (*, *p* < 0.01). Endothelial NOS immunostaining scores (mean ± SD) were 1.50 ± 0.58 in the control group; 1.5 ± 0.58 in the sham group; 4.75 ± 0.50 in the ASP group; 1.25 ± 0.50 in the HT group and 3.75 ± 0.96 in the HT + ASP group.

Figure 2C shows eNOS western blot analysis of the rat aorta. When eNOS protein band intensity was normalized to actin protein band intensity in the same group, it increased significantly in ASP and HT + ASP groups compared to control sham and HT groups (Fig. 2D).

Aortic tissue homogenate nitrite (NO_2_^—^) and nitrate (NO_3_^—^) levels are shown in Fig. 2E. Nitrite and nitrate levels (mean ± SD) in the aorta confirmed significantly increased (*, *p* < 0.05) levels of nitric oxide generation in the ASP group (4.50 ± 1.05 nmol/mg protein) versus control (1.95 ± 0.54 nmol/mg protein), sham (2.78 ± 1.05 nmol/mg protein), HT (0.94 ± 0.33 nmol/mg protein) and HT + ASP (2.22 ± 0.31 nmol/mg protein) groups.

Figure 2F shows rat vessel eNOS mRNA levels. ASP administration significantly increased (*, *p* < 0.05) eNOS mRNA levels compared to control, sham and HT groups. Fold change [2^−(ΔΔct)^] values (mean ± SEM) in eNOS mRNA were 1.23 ± 0.31 in the control group; 1.21 ± 0.27 in the sham group; 19.07 ± 3.49 in the ASP group; 0.90 ± 0.15 in the HT group and 8.60 ± 1.60 in the HT + ASP group.

### Total antioxidant capacity and reactive oxygen–nitrogen species

**Fig. 3:**
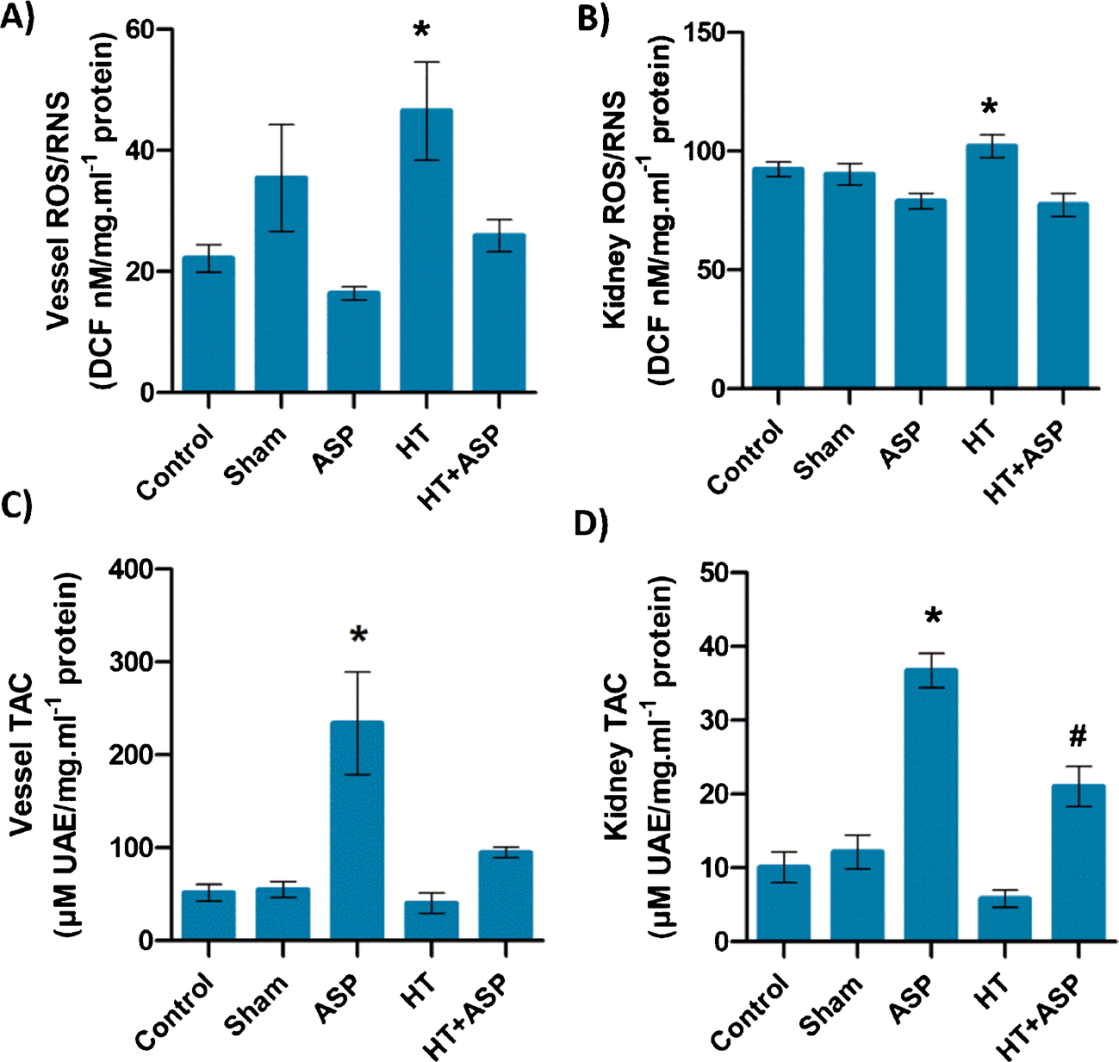
**A-B)** Total reactive oxygen species (ROS) and reactive nitrogen species (RNS). ASP, asperglaucide-treated; HT, hypertension. Data are representative of 8 separate experiments and values are given as mean ± SEM. Statistical analysis of vessel data was done by Kruskal–Wallis One Way Analysis of Variance on Ranks with all pairwise multiple comparison procedures done by Dunn's Method. *, *p* < 0,05 vs. ASP. Statistical analysis of kidney data was done by one-way ANOVA analysis followed by Tukey test. *, *p* < 0,05 vs. ASP and HT + ASP. **C-D)** Total antioxidant capacity (TAC). Data are representative of 8 separate experiments and values are given as mean ± SEM. Statistical analysis was done by one-way ANOVA analysis followed by Tukey test. *, *p* < 0,05 vs. all groups. #, *p* < 0,05 vs. control and HT

Total reactive oxygen/nitrogen species levels and antioxidant capacity in vessel/kidney tissue are shown in Fig. 3. Total reactive oxygen and nitrogen species levels in vessel and kidney tissue were found to be significantly higher (*p < 0.05) in the HT group compared to ASP treated groups (Fig. 3A and B). ASP group had significantly higher TAC in vessel and kidney tissues compared to all other groups (Fig. 3C and D). ASP treatment significantly increased (#*p* < 0.05) TAC in kidney tissue of hypertensive rats compared to the untreated hypertensive group (Fig. 3D).

### Asperglaucide cytotoxicity in HUVEC

**Fig. 4:**
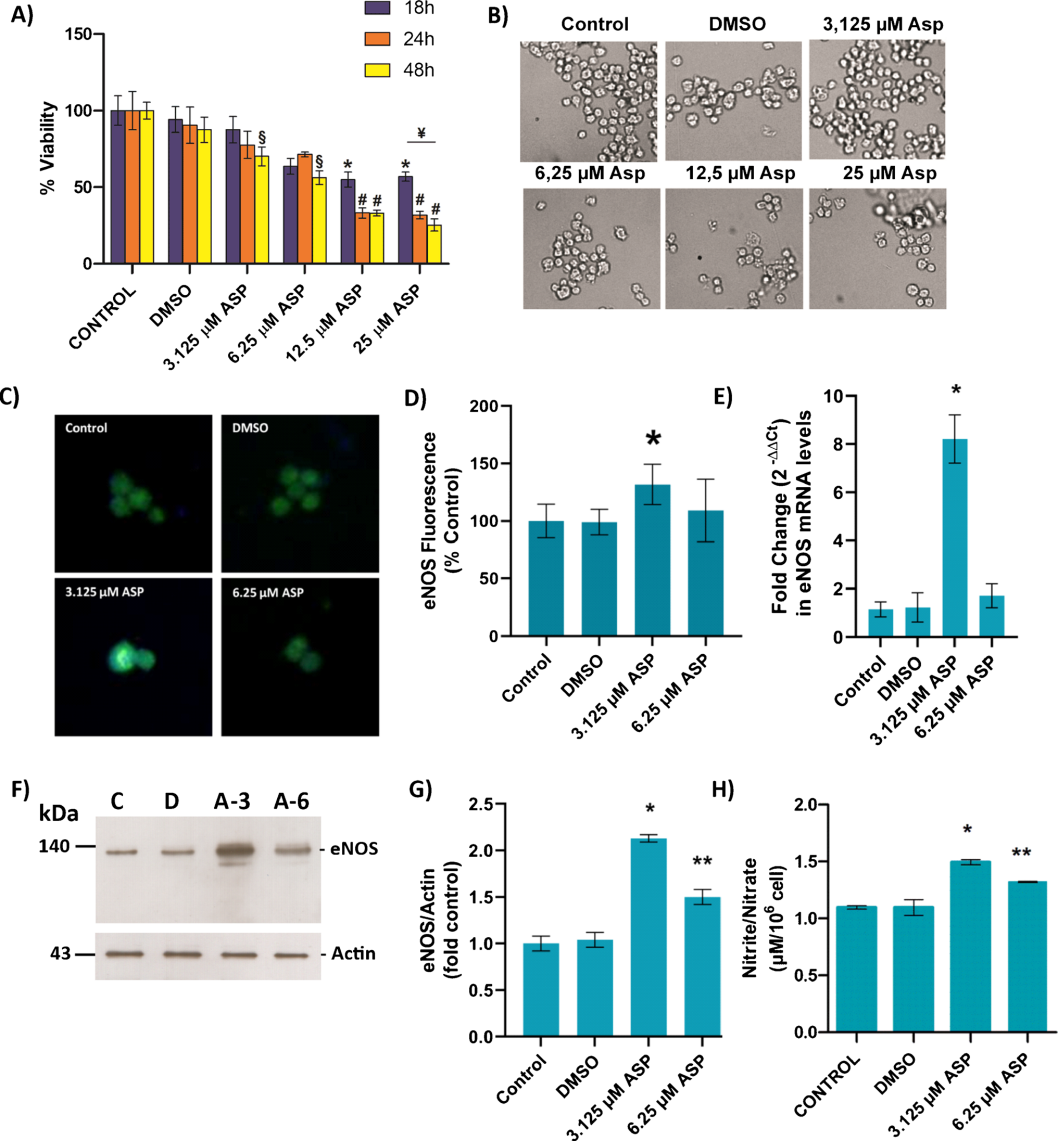
**A)** Cell viability assessed by MTT assay in HUVEC cells. DMSO, cells treated with dimethyl sulfoxide (1 μl/ml); ASP, cells treated with 3.125 µM, 6.25 µM, 12.5 µM and 25 µM asperglaucide. Data shown are representative of 7–8 separate experiments and values are shown as mean ± SEM. Statistical analysis was performed by one-way ANOVA with all pairwise multiple comparison procedures done by Tukey’s multiple comparison test. *p < 0.05, vs. control and DMSO groups at 18 h incubation time. #*p* < 0.05, vs. control, DMSO, 3.125 µM ASP and 6.25 µM ASP groups at 24 h and 48 h incubation time. § < 0.05, vs. control at 48 h incubation time. **B)** Light microscope image (40X magnification) of HUVEC cells treated with ASP at the indicated doses for 18 h. **C)** Representative immunofluorescent staining of eNOS in HUVEC cells. Cells were treated with indicated doses of ASP for 18 h. **D)** Quantitation of fluorescence staining. eNOS staining was quantitated by ImageJ software. Data shown are representative of 6 separate experiments and values are given as mean ± SD. Statistical analysis was performed by one-way ANOVA analysis. Differences between groups were determined by Tukey analysis. *p < 0.05, vs. control and DMSO groups. **E)** HUVEC eNOS mRNA levels. Cells were treated with DMSO (1 μl/ml), 3.125 µM and 6.25 µM ASP for 18 h. Data are representative of 5 separate experiments and values are given as mean ± SEM. Statistical analysis was done by Kruskal Wallis one-way analysis of variance and Tukey's posthoc test was used for comparison between groups. *p < 0.05, compared with control, DMSO, and 6.25 µM ASP groups. **F)** Western blot analysis of eNOS in HUVEC cell lysates. C, control; D, cells treated with dimethyl sulfoxide (1 μl/ml) for 18 h. A-3 and A-6, cells treated with 3.125 µM and 6.25 µM ASP for 18 h, respectively. **G)** The band density of blots was determined by ImageJ software. Data shown are representative of 3 separate measurements and values are given as mean ± SD. Statistical analysis was performed by one way ANOVA and all pairwise multiple comparison procedures done by Tukey test. *p < 0.001, vs. control, DMSO and 6.25 µM ASP groups. ** *p* < 0.001, vs. control, DMSO and 3.125 µM ASP groups. **H)** Nitrite/Nitrate levels in culture medium of HUVEC cells treated with DMSO (1 μl/ml), 3.125 and 6.25 µM ASP. Values are mean ± SEM (*n* = 7–8). Statistical analysis was performed by Kruskal Wallis analysis with all pairwise multiple comparison procedures done by Dunn’s multiple comparison test. *p < 0.05, vs. control and DMSO groups. ** *p* < 0.05, vs. control group

ASP was applied to HUVEC at a dose range of 3.125–25 µM and a dose-dependent 18–48-h cell viability analysis was performed (Fig. 4A). The amount of DMSO applied did not cause toxicity in any time point. Cell groups treated with 12.5 and 25 µM ASP for 18 h showed cytotoxicity compared to the control and DMSO groups (*, *p* < 0.05). A significant decrease in cell viability was observed at 24 and 48 h following 12.5- 25 µM ASP treatment, compared to control, DMSO, 3.125 and 6.25 µM ASP groups (#, *p* < 0.05). In addition, 48 h of 3.125 and 6.25 µM ASP administration significantly decreased cell viability compared to the control group (§, *p* < 0.05). According to these data, ASP was applied for 18 h at non-toxic doses of 3.125 and 6.25 µM.

HUVEC morphology was evaluated under light microscope following 18 h of ASP (3.125–25 µM) treatment. Consistent with the MTT viability data, morphological changes such as shrinkage, clumping, and separation from the surface were observed in cells following 12.5 and 25 µM ASP doses along with a decrease in the cell population (Fig. 4B).

### HUVEC eNOS protein and mRNA levels

Endothelial NOS immunofluorescence staining in HUVEC cells are shown in (Fig. 4C). 3.125 µM ASP application increased eNOS protein levels significantly compared to control, DMSO and 6.25 µM ASP groups (Fig. 4D) (*, *p* < 0.05).

Figure 4E shows eNOS mRNA levels in HUVEC. Application of 3.125 µM ASP significantly increased (*, *p* < 0.05) eNOS mRNA levels compared to control, DMSO and 6.25 µM ASP groups. Fold change [2^−(ΔΔct)^] values (mean ± SEM) in eNOS mRNA were 1.15 ± 0.31 in the control group; 1.23 ± 0.61 in the DMSO group; 8.21 ± 1.00 in the 3.125 µM ASP group and 1.71 ± 0.49 in the 6.25 µM ASP group.

Figure 4F shows eNOS western blot analysis in ASP treated HUVEC. When eNOS protein band intensity was normalized to actin protein band intensity in the same group, it increased significantly (*p* < 0.001) in both 3.125 and 6.25 µM ASP groups compared to control and DMSO groups (Fig. 4G). There was also a significant difference between ASP treatment groups.

Nitrite and nitrate levels in HUVEC cell culture media are shown in Fig. 4H. Nitrite and nitrate levels (mean ± SD) in culture media affirmed significantly increased (*p* < 0.05) levels of nitric oxide generation in the ASP treatment groups. Nitrite and nitrate levels (µM/10^6^cell) were 1.10- ± 0.04 in the control group; 1.10 ± 0.20 in the DMSO group; 1.49 ± 0.06 in the 3.125 µM ASP group and 1.32 ± 0.02 in the 6.25 µM ASP group.

## Discussion

The renovascular 2K1C hypertension model used herein exhibits hemodynamic and structural changes of hypertension and is an equivalent model of renal artery stenosis in humans. The decrease of renal blood flow owing to renal artery stenosis can be caused by obstructive vascular diseases, such as atherosclerosis [21]. We observed that after unilateral renal artery stenosis, blood pressure rose rapidly in rats and reached a hypertensive plateau within two weeks. Hypertension was maintained throughout the study period, from the first to the 4th week. Our findings are in accord with other studies reporting increased blood pressure four weeks after clipping [40]. Likewise, many studies have also shown an increase in weight of the contralateral kidney while the weight of the clipped kidney decreased with time [23]. In the surgical hypertension model used, the kidney, whose blood flow decreases, secretes renin, which causes increased angiotensin II (ANG II) generation and as a result raises blood pressure. As blood pressure rises, sodium excretion by the undamaged contralateral kidney increases, a term called pressure natriuresis; hence there is no sodium retention [26]. Prostanoids, which are vasoactive mediators, are also implicated in creating hypertension in the 2K1C model [56]. We observed that compensative hypertrophy of the contralateral kidney was attenuated in the HT + ASP group compared with that in the HT group. This indicates that ASP treatment attenuated inflammation and fibrosis reported to be activated in the contralateral kidney after the initiation of renal artery stenosis. As a result, ASP may restrain compensative hypertrophy of the contralateral kidney by the inhibition of fibrosis and inflammation.

As stated in the introduction, ASP is a dipeptide consisting of N-benzoylphenyalanine and phenyalanine. Dipeptides Ile-Arg, Lys-Phe and Glu-Phe were found to inhibit renin activity [28]. Quantitative structure–activity relationship (QSAR) using the peptide dataset indicated that the existence of N-terminal aliphatic residues such as isoleucine, leucine, valine and C-terminal large amino acid residues such as phenylalanine, tryptophan led to higher renin inhibitory activity of dipeptides [45]. Shifting the arrangement of amino acids residues from Thr-Phe to Phe-Thr resulted in a considerable decrease in activity, signifying the vital role of the C-terminal bulky hydrophobic moiety to renin inhibition [45]. Future studies are needed to determine the effect of ASP on renin activity.

The selected ASP dose and method of preparation were determined according to previous studies [42]. ASP is aurantiamide acetate, and the distribution of aurantiamide acetate in different rat organs were investigated following the administration of purslane extract containing 0.2 mg/kg aurantiamide acetate [14]. The distribution of aurantiamide acetate occured rapidly in rats, ensuing a widespread delivery of the compound to different organs. Aurantiamide actetate increased in the heart and kidney, reaching a peak level at about 30 min after administration. The concentrations of aurantiamide acetate in the spleen, liver, small intestine and brain decreased with time and after 4 h, was reduced by about 90% [14]. These results suggest that aurantiamide acetate results in no long-term accumulation in rat tissues. Indeed, we have observed no toxicity of ASP at the administered dose 0.5 mg/kg/day. Body weight measured after the 4th week showed a significant increase in all experimental groups, and there was no significant difference in body weight among the experimental groups. Likewise, serum biochemical profile determined after the 4th week showed no significant difference between experimental groups.

Increased peripheral vascular resistance and impaired vascular reactivity fulfill important roles in the pathogenesis of hypertension. In the present study, vascular reactivity of thoracic aorta was investigated in ASP treated of hypertensive rats. Vascular constriction–relaxation responses and vascular sensitivity to constrictor and dilator stimuli were assessed using KCl, Phe, ACh, and SNP. As predicted, Phe-mediated constriction response was found to be increased in the aortas of hypertensive animals compared with controls. This finding is in agreement with previous studies performed on renovascular models of hypertension [22, 33]. Increased responsiveness to Phe may be linked to decreased endothelium-derived relaxing factors such as nitric oxide (NO) [25] or increased contracting factors such as thromboxane A2, prostaglandin H2 and endoperoxides [18]. We observed decreased and unchanged vasorelaxation response to Ach and SNP, respectively. Acetylcholine vasorelaxant action is mediated by muscarinic M3 receptor activation which leads to the release of NO from endothelial cells [50]. Vasorelaxation response to Ach in isolated aortic rings has therefore been considered as an indication of the status of NO release and endothelium-dependent relaxation, whereas that of SNP has been used as evidence of the status of endothelium-independent relaxation [5]. Once produced by eNOS, NO diffuses to close by smooth muscle cells, where it reacts with the ferrous iron of the heme group of guanylate cyclase, causing synthesis of cyclic guanosine monophosphate (cGMP) from guanosine triphosphate (GTP). Cyclic GMP then stimulates protein kinase G (PKG), which in turn triggers Ca^2+^-ATPase-dependent refilling of intracellular calcium stores and lowering of intracellular calcium levels, thus mediating smooth muscle cell relaxation [6]. ASP was found to significantly lower blood pressure in hypertensive rats 1 week after treatment by increasing endothelium-mediated relaxation response. ASP treatment also resulted in reduced Phe-mediated contractile responses, which were increased in the hypertension group. The presented data shows that antihypertensive effects of ASP may partly be due to increased eNOS protein expression in 2K1C hypertensive rats.

The mechanism of ASP-induced eNOS expression in the rat model of renovascular arterial hypertension is likely to be through suppression of tumor necrosis factor-alpha (TNF-alpha) expression. Tumor necrosis factor-alpha is a key proinflammatory cytokine mediating angiotensin II-induced renovascular (2K1C) hypertension [27]. TNF-alpha is elevated in the plasma of 2K1C hypertensive rats [10] and the induced increase in blood pressure in 2K1C animals is significantly reduced by nanoinjection of anti-TNF-alpha type-1 receptor (TNFR1) antibody [27]. Tumor necrosis factor-alpha decreases eNOS mRNA levels [39]. Yoshizumi et al. showed that there was a striking decrease in steady-state levels of eNOS mRNA and protein in human umbilical vein endothelial cells (HUVECs) treated with TNF-alpha [54]. This discovery is consistent with earlier studies presenting impaired endothelium dependent vasorelaxation in isolated arteries treated with TNF-alpha [4]. Tumor necrosis factor-alpha-induced destabilization of eNOS mRNA has also been reported by others [32].

Metabolites from aurantiamide acetate have been shown to reduce lipopolysaccharide (LPS)-induced TNF-alpha levels in mouse microglial cells [53]. Likewise, aurantiamide acetate was reported to decrease TNF-alpha levels in whole blood of rat [38] and in LPS-induced RAW264.7 cells [47]. A more recent study has also revealed that aurantiamide acetate significantly suppressed TNF-alpha production in LPS-induced acute lung injury in mice [19].

Compounds that increase eNOS protein levels are only favorable when ensuring eNOS functionality [29]. An approach to increase eNOS protein without a concurrent elevation of the essential NOS cofactor (6R-)5,6,7,8-tetrahydrobiopterin may lead to eNOS uncoupling and oxidative stress [20]. Previous studies have identified compounds that maintain eNOS functionality in disease, and at the same time, upregulate expression of the enzyme [51]. Future studies can further elucidate the beneficial effect of ASP as a small-molecular-weight compound enhancing eNOS expression.

Red blood cell deformability is an important parameter affecting the fluidity of blood. Deformability of erythrocytes is a significant factor often used for hemorheological evaluations in experimental and clinical studies. Erythrocyte deformability is defined as the ability to change shape in response to forces acting on red blood cells during flow and provides the ability for erythrocytes to traverse capillaries smaller than their resting diameter [15]. Decreased red blood cell deformability are reported hemorheological changes in patients with hypertension [2]. In view of the fact that the diameter of the red blood cell is approximately 7.5 to 8.7 µm and that of the smallest blood vessels is around 5–7 µm [17], the capability of the cell to deform is very important for capillary flow, and a decreased erythrocyte deformability could cause an increased microvascular flow resistance [37]. Mchedlishvili et al. proposed that alterations in red blood cell mechanical properties lead to increased blood pressure and such changes may play a role in the increased peripheral vascular resistance [30]. A major determining factor of red cell deformability is the activity of Na,K-ATPase as it maintains the electrochemical gradient through the cell membrane [36]. Na,K-ATPase appears to be mostly responsible for alterations in RBC deformability in patients suffering from hypertension [35]. A previous study reported a significant decrease in red blood cell transit times in hypertensive rat samples incubated with SNP, implying increased deformability [11]. We have also observed increased erythrocyte deformability in hypertensive rats treated with ASP. This effect was observed only in hypertensive rat samples and can be regarded as the correction of the deterioration. The in vivo effects of ASP resulting in increased nitric oxide production and RBC deformability observed in hypertensive rats might be expected to improve blood fluidity and decrease flow resistance. It appears that increased extracellular nitric oxide also influences red blood cell structural and functional properties as reported previously [41].

Increased ANG II formation in the 2K1C renovascular hypertension model activates NAD(P)H oxidase, a major source for reactive oxygen species (ROS) generation in vascular tissues, causing an imbalance between pro- and antioxidant mechanisms [49]. ASP-mediated reduction in the blood pressure of 2 K-1C rats may be related to its antioxidant properties [42]. Antioxidants have been demonstrated to reduce blood pressure in 2K1C hypertensive rats associated with oxidative stress [12].

The cytotoxic activity of aurantiamide acetate was determined by MTT on in vitro cultured HUVEC. Aurantiamide acetate decreased cell viability in a dose- and time-dependent manner. A significant decrease in cell viability was observed at 24 h following 12.5- 25 µM ASP treatment compared to control, which agrees with a study which demonstrated that 24 h incubation of 10 µM aurantiamide acetate, significantly decreased the viability of human malignant glioma U87 [53]. ASP treatment at noncytotoxic 3 µM dose was found to significantly increase eNOS gene expression and protein levels in human endothelial cells which is in accord with data obtained from in vivo experiments performed on rats.

In conclusion, ASP administration improved endothelial-dependent vascular relaxation and reduced vascular oxidative stress in a 2 K-1C renovascular hypertension model. Administration of ASP significantly increased eNOS gene expression and blood flow in hypertensive rats. At non-cytotoxic doses, ASP significantly increased levels of eNOS protein in human endothelial cells. ASP may have potential as an anti-hypertensive agent in future studies.

## Supplementary Information

Below is the link to the electronic supplementary material.

Supplementary file1 (PNG 5947 kb)

High resolution image (TIF 38485 kb)

Supplementary file2 (PNG 118 kb)

High resolution image (TIF 10244 kb)

Supplementary file3 (DOCX 14 kb)

Supplementary file4 (DOCX 32 kb)

## Data Availability

Data that support the findings of this study are available on request from the corresponding author, MA.

## Abbreviations

2 K-1C: Two-kidney one-clip
Ach: Acetylcholine
ALB: Albumin
ALT: Alanine aminotransferase
ANOVA: Analysis of variance
ASP: Asperglaucide
AST: Aspartate aminotransferase
BUN: Blood urea nitrogen
C: Control
cGMP: Guanosine monophosphate
CRE: Creatinine
Ct: Threshold cycle
CTCF: Corrected total cell fluorescence
HT: Hypertensive
HUVEC: Human umbilical vein endothelial cells
iNOS: Inducible nitric oxide synthase
LPS: Lipopolysaccharide
MBP: Mean blood pressure
MTT: 3-(4,5-Dimethylthiazol-2-yl)-2,5-diphenyl tetrazolium bromide
NO: Nitric oxide
NO_2_^—^: Nitrite
NO_3_^—^: Nitrate
OMIM: Online mendelian inheritance in man
Phe: Phenylephrine
PKG: Protein kinase G
QSAR: Quantitative structure–activity relationship
RBC: Red blood cell
ROS: Reactive oxygen species
Sham: Sham operated
SNP: Sodium nitroprusside
TBIL: Total bilirubin
