# Supplementary figures and images for: Effects of aurantiamide on a rat model of renovascular arterial hypertension
Source: Pflugers Arch. 2023 Aug 15;475(10):1177–92. doi: 10.1007/s00424-023-02850-8 (PMC10499692; doi:10.1007/s00424-023-02850-8)

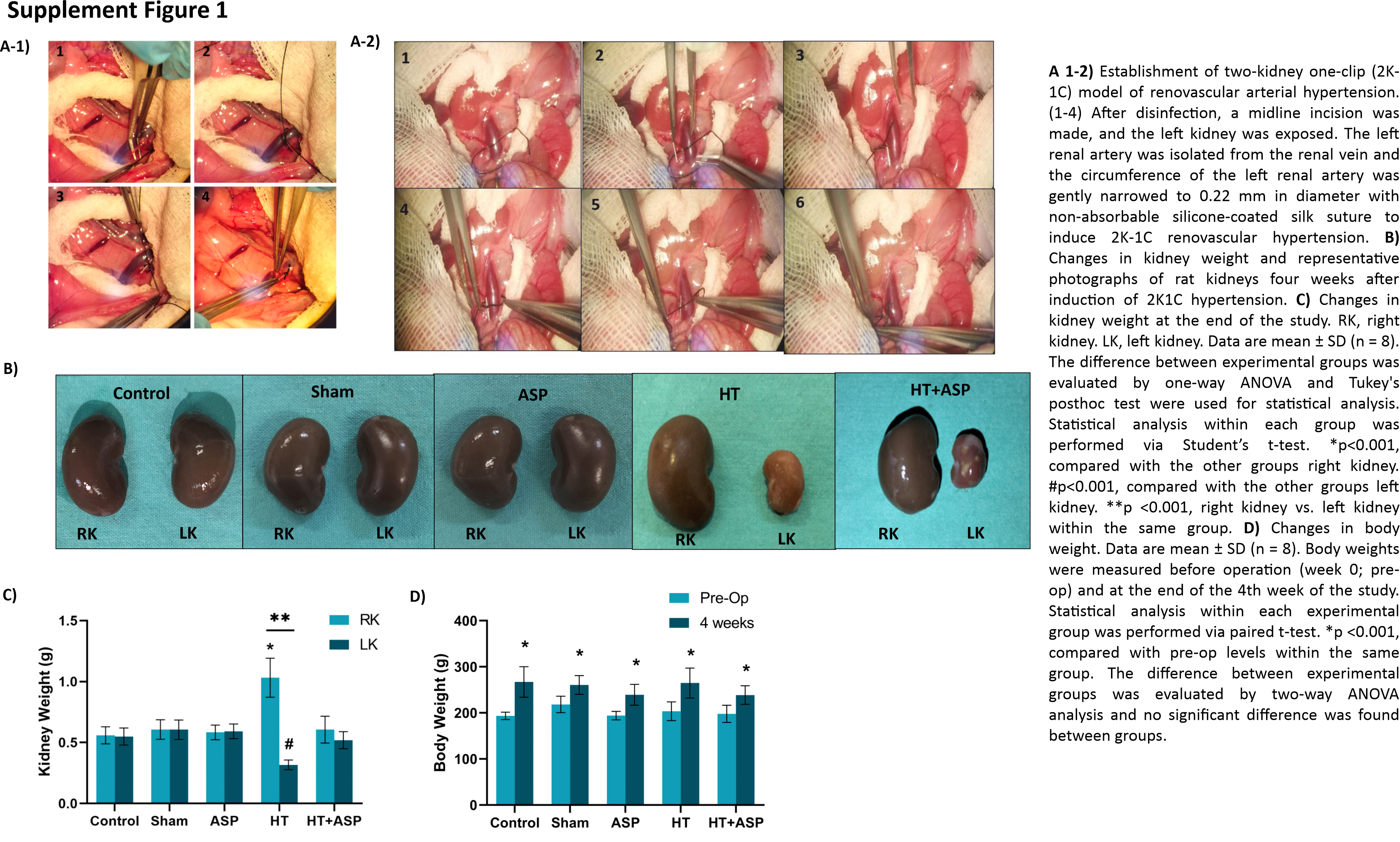

Supplement: Supplementary file 1 — Supplementary file1 (PNG 5947 kb) [file 424_2023_2850_Fig5_ESM.png]

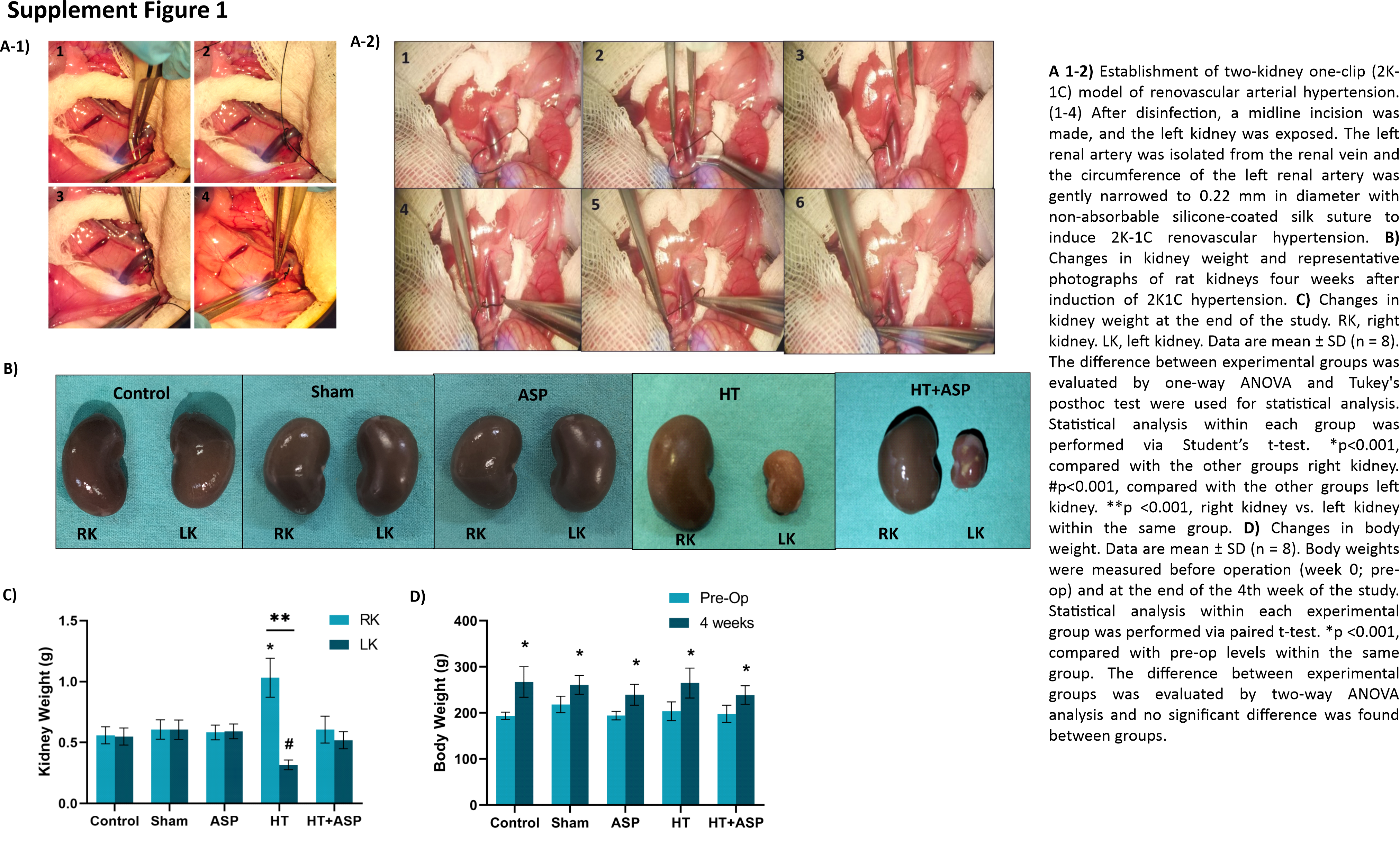

Supplement: Supplementary file 2 — High resolution image (TIF 38485 kb) [file 424_2023_2850_MOESM1_ESM.tif]

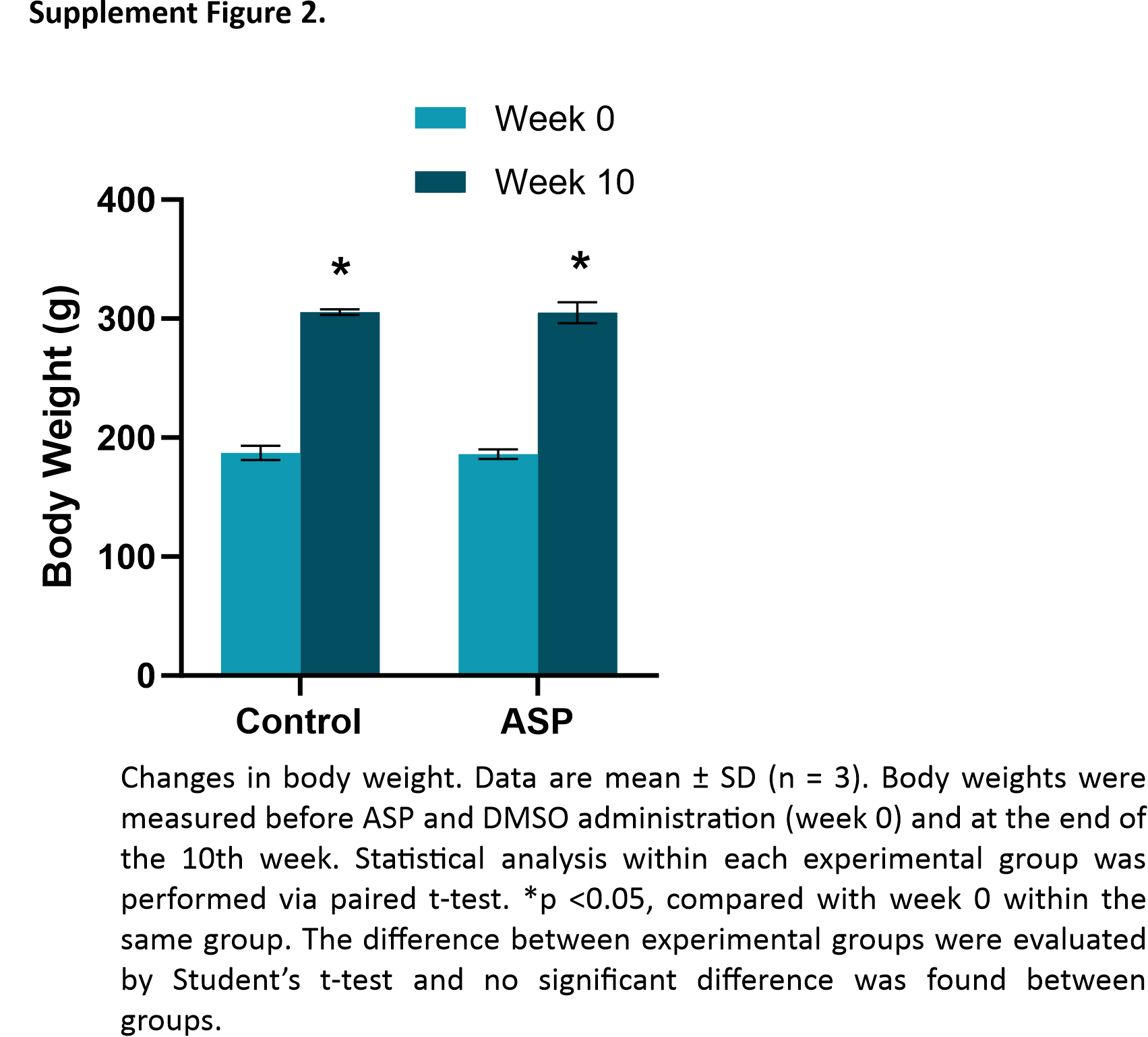

Supplement: Supplementary file 3 — Supplementary file2 (PNG 118 kb) [file 424_2023_2850_Fig6_ESM.png]

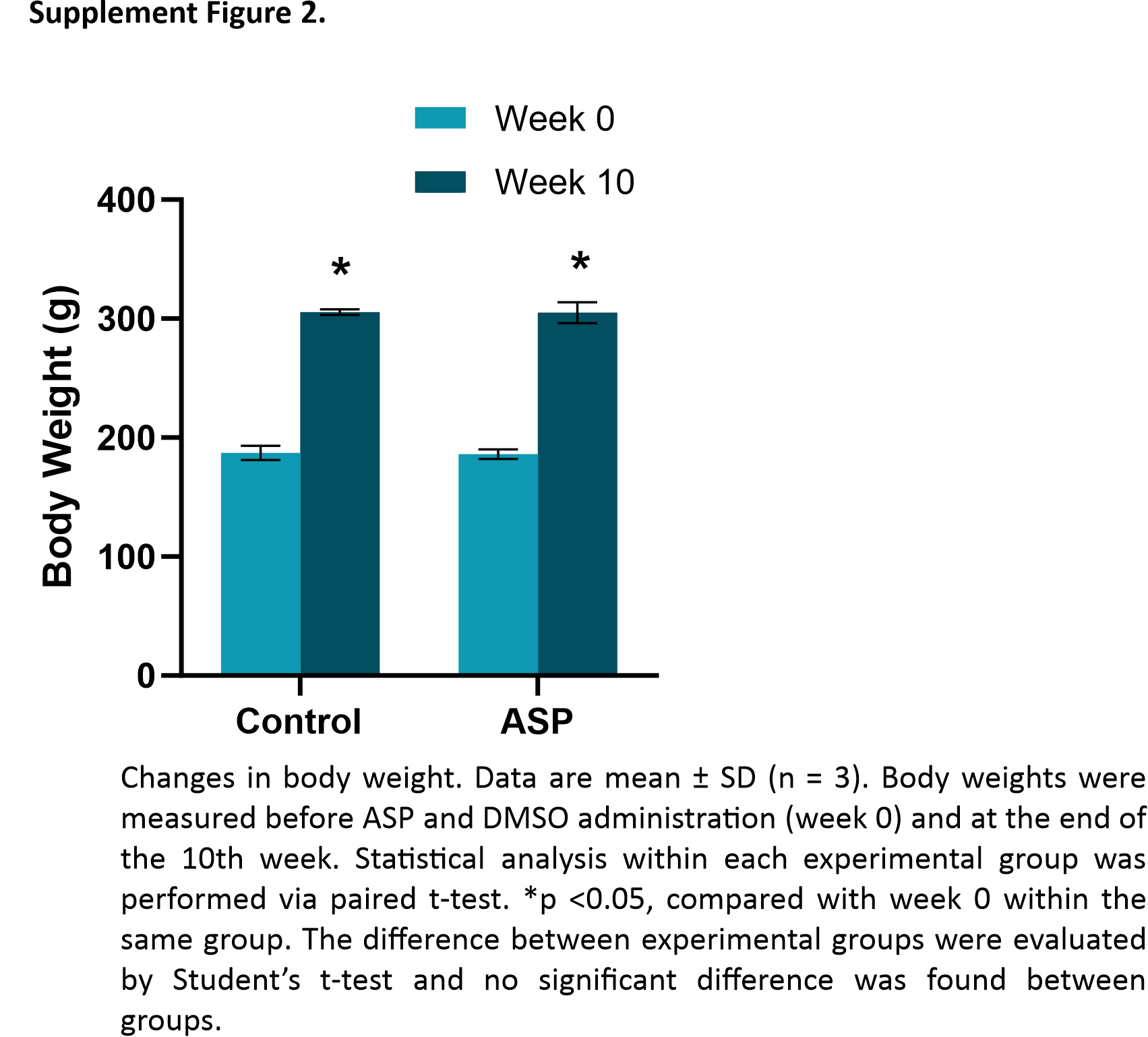

Supplement: Supplementary file 4 — High resolution image (TIF 10244 kb) [file 424_2023_2850_MOESM2_ESM.tif]
