# Supplementary material for: Effects of aurantiamide on a rat model of renovascular arterial hypertension
Source: Pflugers Arch. 2023 Aug 15;475(10):1177–92. doi: 10.1007/s00424-023-02850-8 (PMC10499692; doi:10.1007/s00424-023-02850-8)
Supplement: Supplementary file 5 — Supplementary file3 (DOCX 14 kb) [file 424_2023_2850_MOESM3_ESM.docx]

**Supplement Table 1**. **Laboratory values measured at 10th week.**

| **Variable** | **Control (n=3)** | **ASP (n=3)** |
| --- | --- | --- |
| **ALT (U/l)** | 44.00 ± 2.65 | 46.67 ± 0.58 |
| **ALB (g/dl)** | 3.93 ± 0.06 | 4.00 ± 0.10 |
| **TBIL (mg/dl)** | 0.20 ± 0.00 | 0.23 ± 0.06 |
| **CRE (mg/dl)** | 0.20 ± 0.00 | 0.20 ± 0.00 |
| **BUN (mg/dl)** | 28.33 ± 0.97 | 27.87 ± 0.45 |
| Values given as mean ± SD. ALT, alanine aminotransferase; ALB, albumin; TBIL, total bilirubin; CRE, creatinine; BUN, blood urea nitrogen. Statistical analysis was performed by Student's t-test and no significant difference was found between groups. | | |
