# Supplementary material for: Effects of aurantiamide on a rat model of renovascular arterial hypertension
Source: Pflugers Arch. 2023 Aug 15;475(10):1177–92. doi: 10.1007/s00424-023-02850-8 (PMC10499692; doi:10.1007/s00424-023-02850-8)
Supplement: Supplementary file 6 — Supplementary file4 (DOCX 32 kb) [file 424_2023_2850_MOESM4_ESM.docx]

**Supplement Table 2**. **Cell viability assessed by MTT assay in HUVEC cells.**

DMSO, cells treated with dimethyl sulfoxide (1μl/ml); ASP, cells treated with 3.125 µM, 6.25 µM, 12.5 µM and 25 µM asperglaucide. Data shown are representative of 7-8 separate experiments.
